# A Series of Anatomical Plates in Lithography, with References and Physiological Comments, Illustrating the Structure of the Different Parts of the Human Body

**Published:** 1834-04-01

**Authors:** 


*A Series of Anatomical Plates in Lithography, with References and Physiological Comments, illustrating the Structure of the different Parts of the Human Body.* Edited by Jones Quain, m.d., Professor of Anatomy and Physiology in the University of London. Fasciculi 5, 6, 8, 9, 10.

These are correct as well as spirited drawings, though we cannot help regretting that Dr. Quain should have thought it expedient to publish his work in such exceedingly minute fragments. We should advise anatomical students, however, to rectify this mistake of the learned professor by their method of purchasing his book, taking it in by divisions rather than by fasciculi.

